# Supplementary material for: Phenolic Profile, Antioxidant and Enzyme Inhibition Properties of the Chilean Endemic Plant Ovidia pillopillo (Gay) Meissner (Thymelaeaceae)
Source: Metabolites. 2022 Jan 18;12(2):90. doi: 10.3390/metabo12020090 (PMC8876944; doi:10.3390/metabo12020090)
Supplement: Supplementary file 1 [file metabolites-12-00090-s001.zip › metabolites-1520556-supplementary1.18.pdf]

Supplementary material for the Article

Phenolic Profile, Antioxidant and Enzyme Inhibition Properties of the Chilean Endemic Plant *Ovidia pillopillo* (Gay) Meissner (Thymelaeaceae)

Carmen Cortés [1,†], Diego A. González-Cabrera [1,†], Ruth Barrientos [1], Claudio Parra [2], Javier Romero-Parra [3], Mariano Walter Pertino [4,*], Carlos Areche [5], Beatriz Sepúlveda [6], Jorge Bórquez [7], Alfredo Torres-Benítez [1] and Mario J. Simirgiotis [1,*]

[1] Instituto de Farmacia, Facultad de Ciencias, Campus Isla Teja, Universidad Austral de Chile, Valdivia 5090000, Chile; carmenc1012@gmail.com (C.C.); diego.gonzalez@alumnos.uach.cl (D.A.G.-C.); ruth.barrientos@alumnos.uach.cl (R.B.); aljotobe19@hotmail.com (A.T.-B.)
[2] Laboratorio de Química Orgánica y Productos Naturales, Facultad de Ciencias Agronómicas, Universidad de Tarapacá, Av. General Velásquez 1775, Arica 1000000, Chile; cparra@uta.cl
[3] Departamento de Química Orgánica y Fisicoquímica, Facultad de Ciencias Químicas y Farmacéuticas, Universidad de Chile, Olivos 1007, Casilla 233, Santiago 6640022, Chile; javier.romero@ciq.uchile.cl
[4] Laboratorio de Química de Productos Naturales, Instituto de Química de Recursos Naturales, Universidad de Talca, Talca 3460000, Chile
[5] Departamento de Química, Facultad de Ciencias, Universidad de Chile, Las Palmeras 3425, Nuñoa, Santiago 7800024, Chile; areche@uchile.cl
[6] Departamento de Ciencias Químicas, Universidad Andres Bello, Campus Viña del Mar, Quillota 980, Viña del Mar 2520000, Chile; bsepulveda@uc.cl
[7] Departamento de Química, Facultad de Ciencias Básicas, Universidad de Antofagasta, Antofagasta 1240000, Chile; jorge.borquez@uantof.cl
* Correspondence: mwalter@utalca.cl (M.W.P.); mario.simirgiotis@uach.cl (M.J.S.); Tel.: +56-999835427 (M.J.S.)
† These authors contributed equally to this work.

Citation: Cortés, C.; González-Cabrera, D.; Barrientos, R.; Parra, C.; Romero Parra, J.; Pertino, M.W.; Areche, C.; Sepúlveda, B.; Bórquez, J.; Torres-Benítez, A.; et al. Phenolic Profile, Antioxidant and Enzyme Inhibition Properties of the Chilean Endemic Plant *Ovidia pillopillo* (Gay) Meissner (Thymelaeaceae). *Metabolites* **2022**, *11*, 90. https://doi.org/10.3390/12020090

Academic Editor: Gabriele Capodaglio

Received: 6 December 2021
Accepted: 17 January 2022
Published: 18 January 2022

Publisher's Note: MDPI stays neutral with regard to jurisdictional claims in published maps and institutional affiliations.

Copyright: © 2022 by the authors. Submitted for possible open access publication under the terms and conditions of the Creative Commons Attribution (CC BY) license (https://creativecommons.org/licenses/by/4.0/).

Figure S1: (**a–e**) Full MS spectra and structures of compounds detected in *O. pillo- pillo* tincture (peaks 10, 13, 14, 15 and 17).

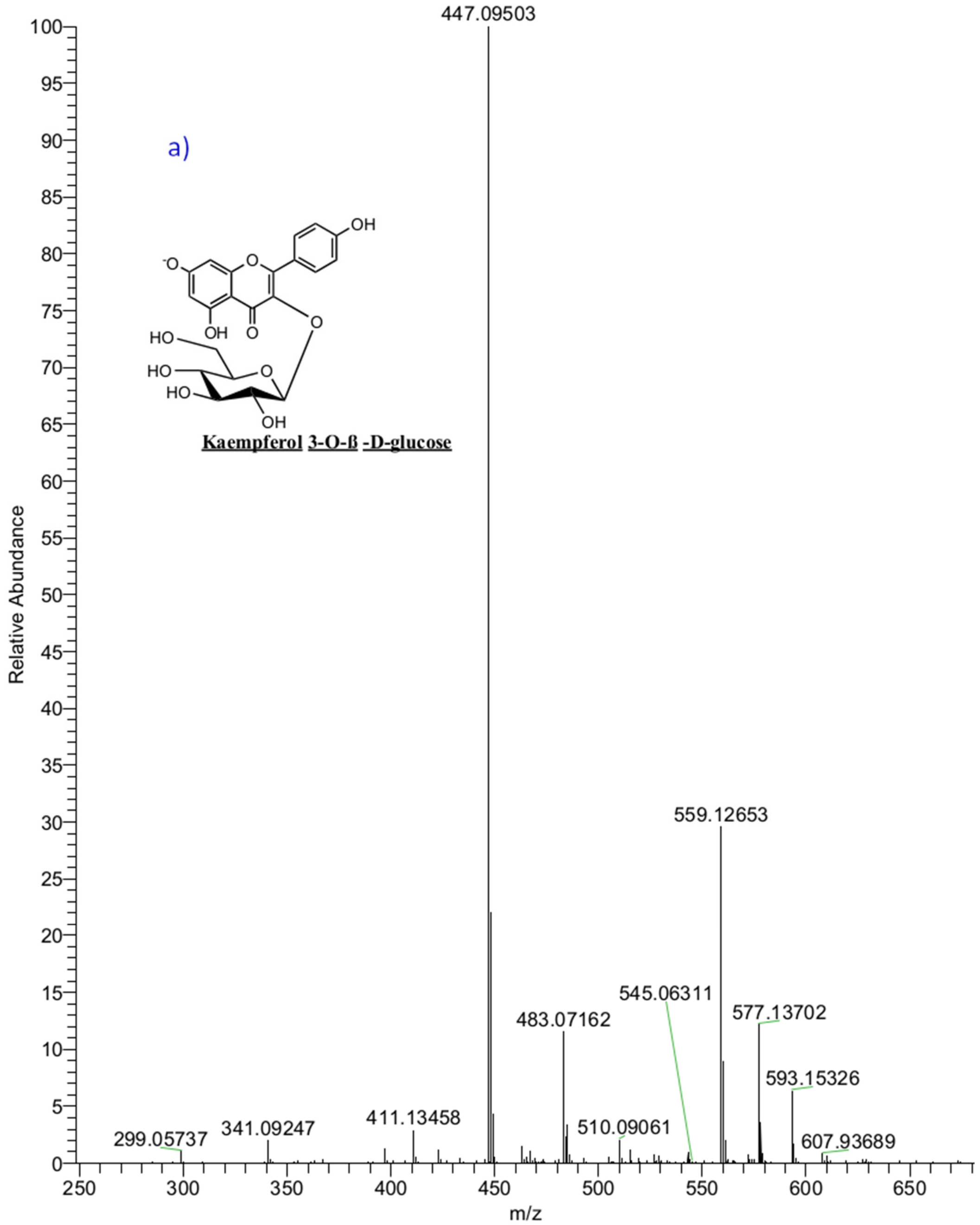

a)
Kaempferol 3-O-ß -D-glucose
447.09503
559.12653
545.06311
577.13702
483.07162
593.15326
411.13458
510.09061
299.05737
341.09247
607.93689
Relative Abundance
m/z

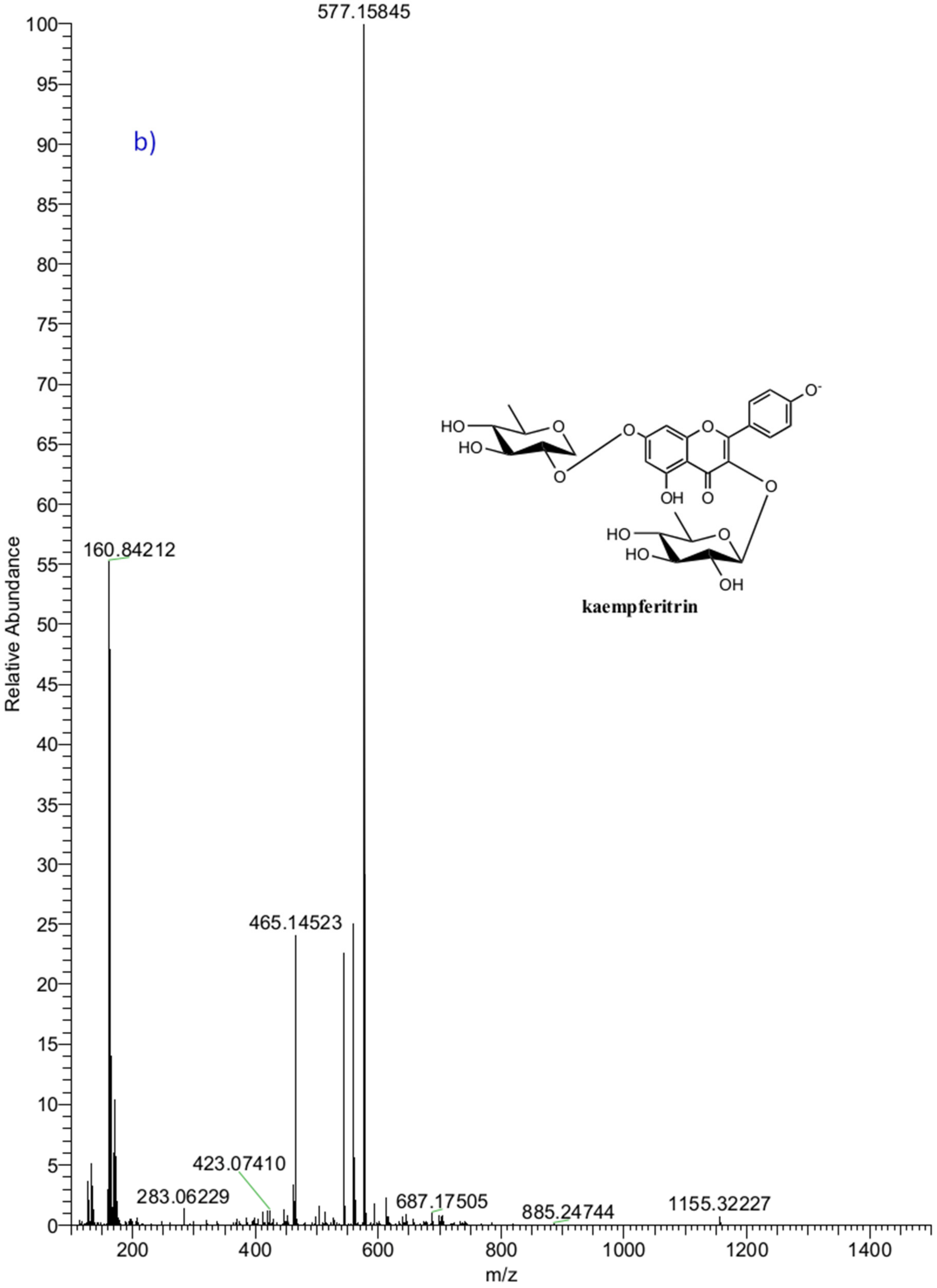
b)
577.15845
160.84212
465.14523
423.07410
283.06229
687.17505
885.24744
1155.32227
Relative Abundance
m/z
kaempferitrin

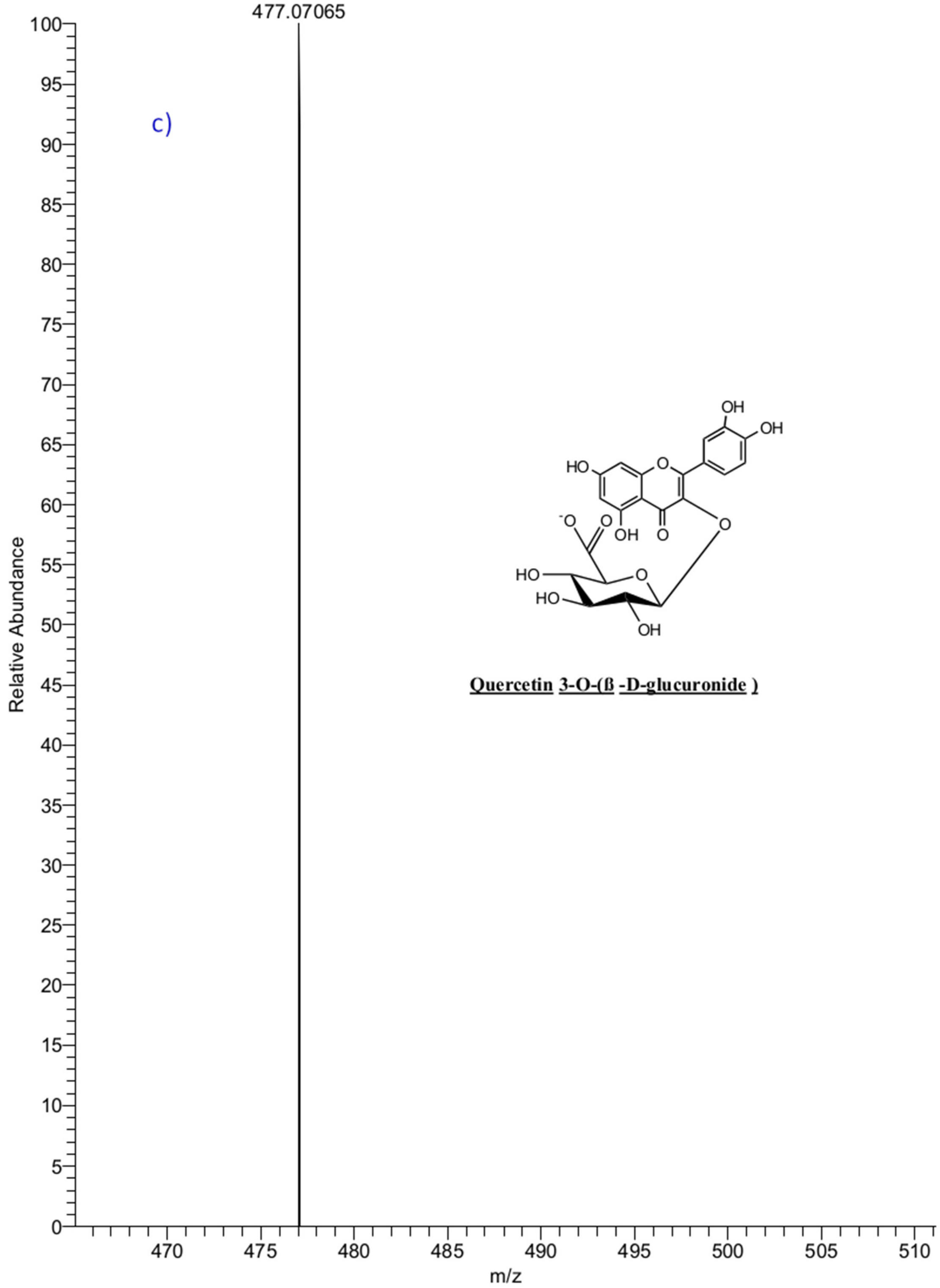

Quercetin 3-O-(ß -D-glucuronide)

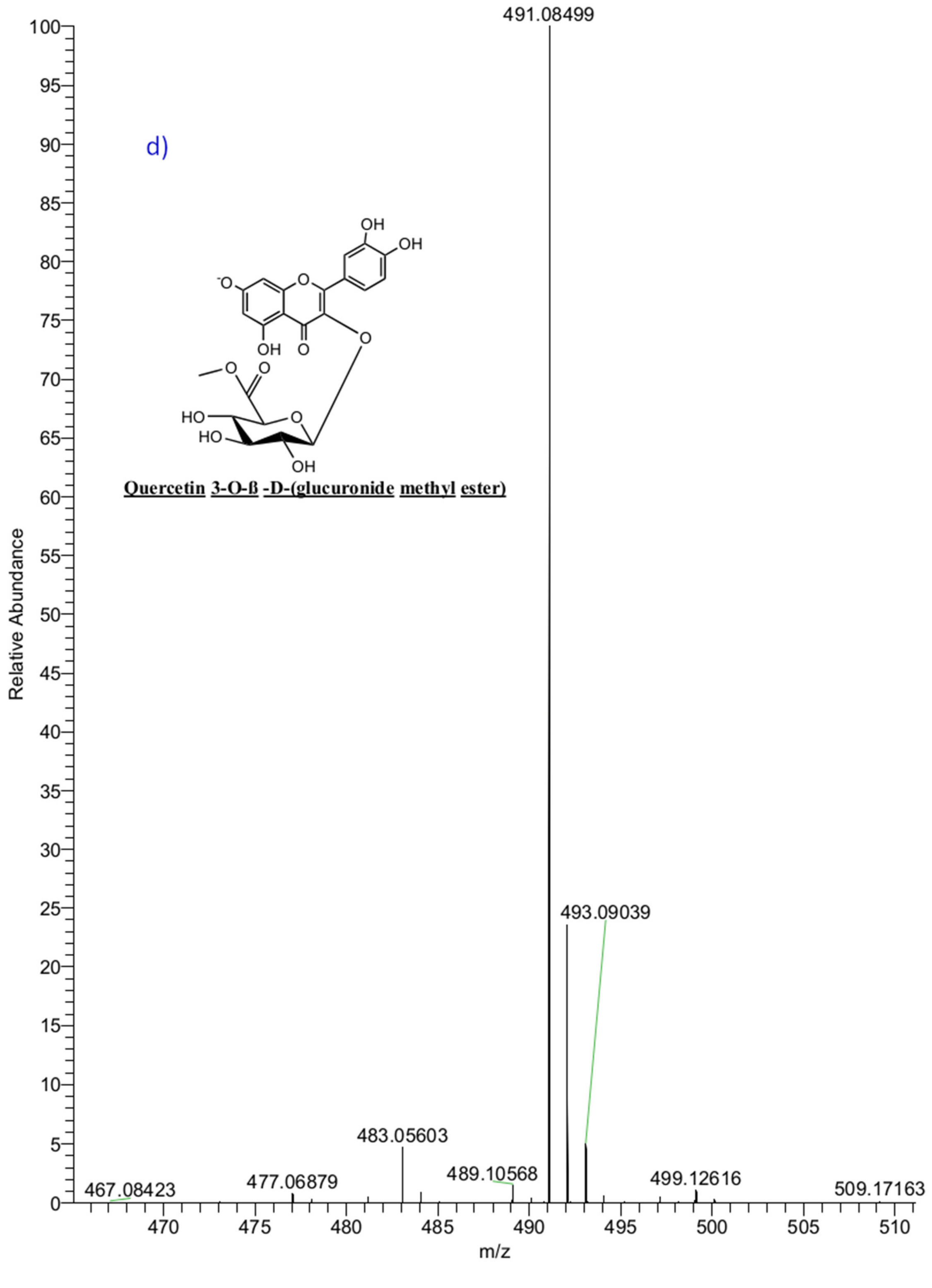

d)
Quercetin 3-O-ß -D-(glucuronide methyl ester)
491.08499
493.09039
483.05603
489.10568
477.06879
467.08423
499.12616
509.17163
Relative Abundance
m/z

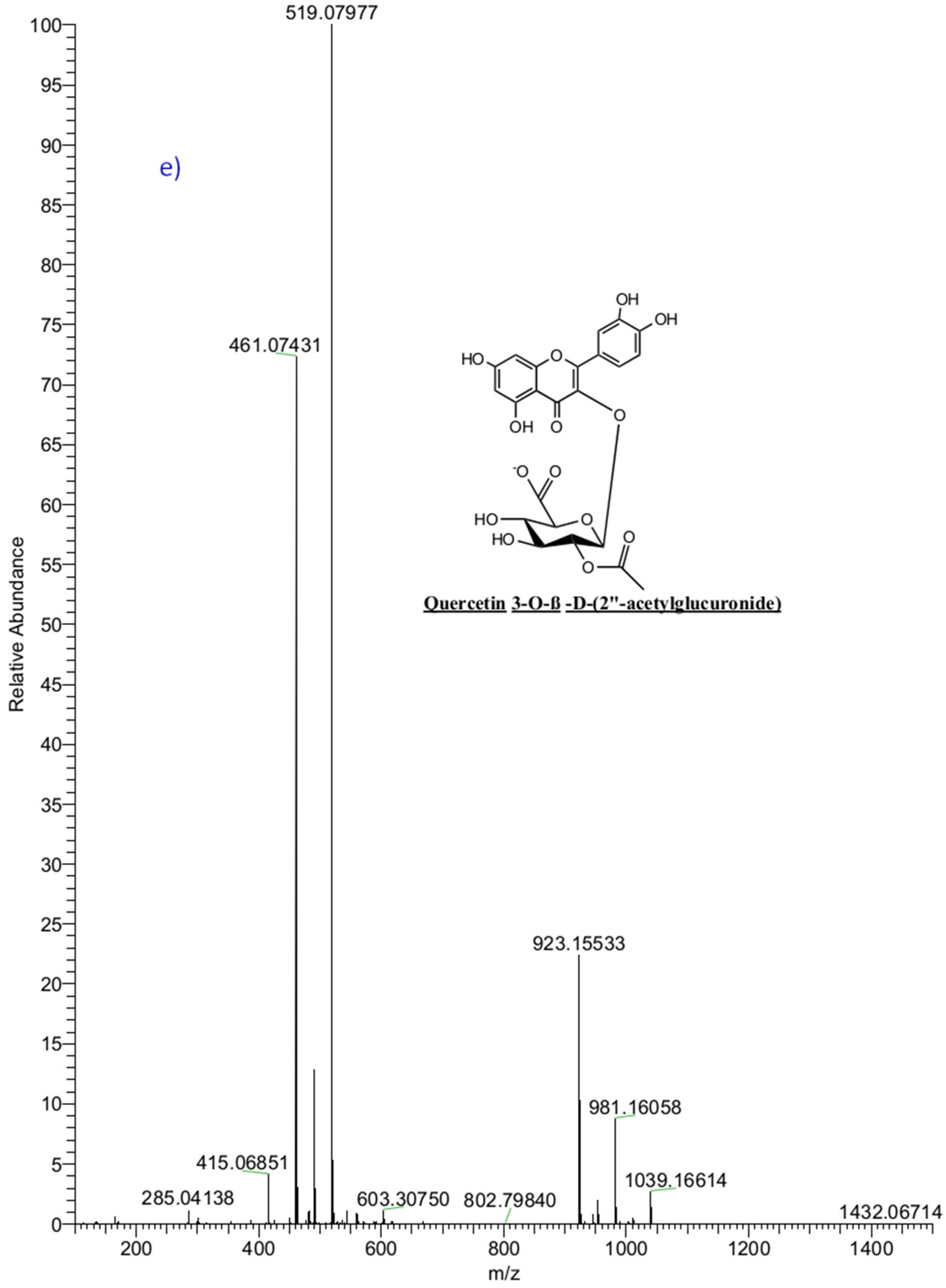

e)
519.07977
461.07431
923.15533
981.16058
415.06851
1039.16614
285.04138
603.30750
802.79840
1432.06714
Relative Abundance
m/z
HO
OH
O
Quercetin 3-O-ß -D-(2''-acetylglucuronide)

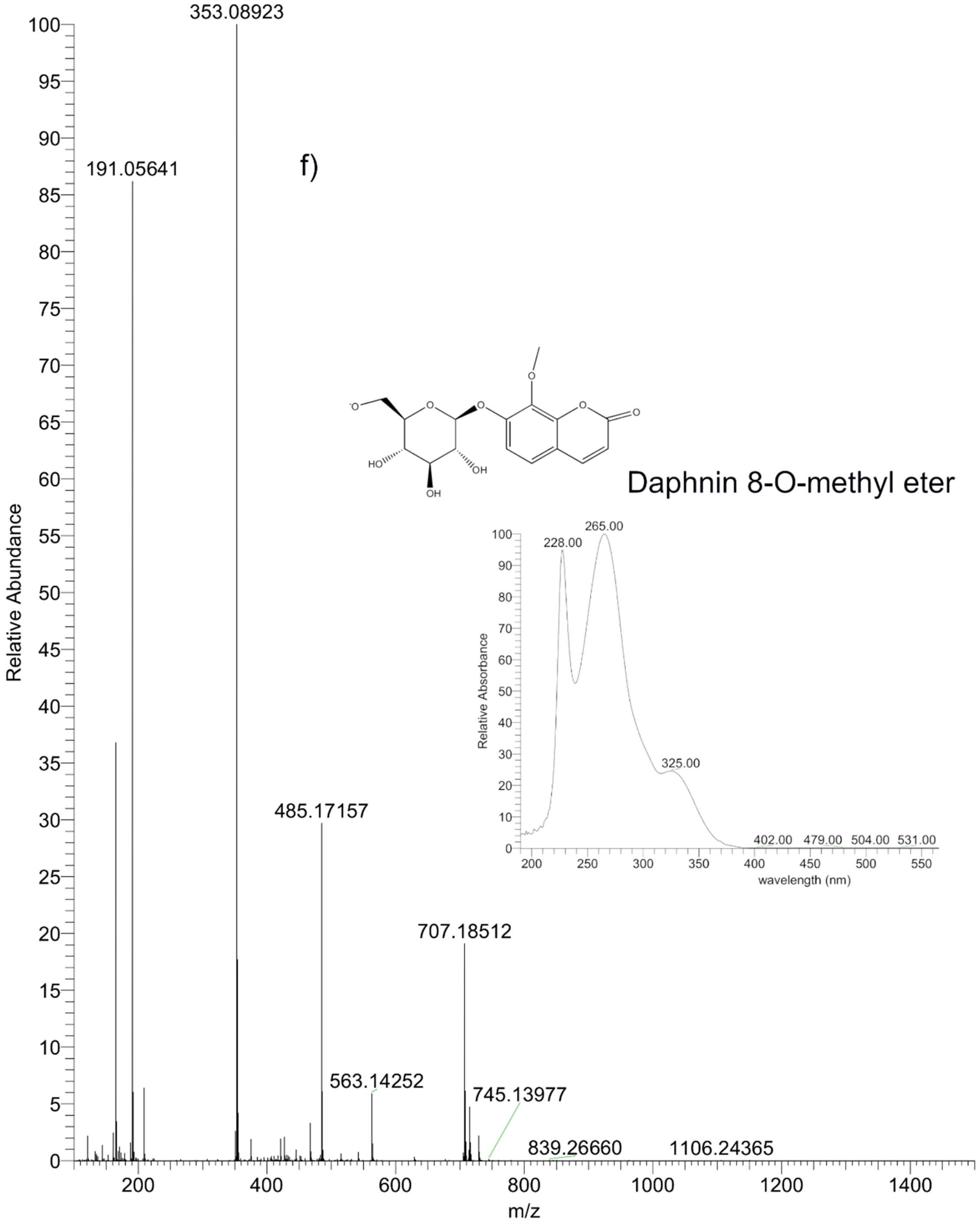
f)
353.08923
191.05641
485.17157
707.18512
563.14252
745.13977
839.26660
1106.24365
Relative Abundance
m/z
Daphnin 8-O-methyl eter
228.00
265.00
325.00
402.00
479.00
504.00
531.00
Relative Absorbance
wavelength (nm)
